# Intravitreal methotrexate in type B lymphoblastic leukemia—Case Report

**DOI:** 10.3389/fonc.2023.1237204

**Published:** 2023-08-28

**Authors:** Maria Isabel Maya Naranjo, Sebastián Vallejo López, Sara Mesa Mesa, Mónica Ortiz Pérez, Mariana López Posada, Martha Lía Gaviria Bravo, María Elena González Alviar

**Affiliations:** ^1^ Department of Ophthalmology, Universidad Pontificia Bolivariana, Medellín, Colombia; ^2^ Department of Ophthalmology, Universidad de Antioquia, Medellín, Colombia

**Keywords:** methotrexate, acute lymphoblastic leukemia, intravitreal injection, case report, hypopion

## Abstract

Leukemia is a common neoplasia that, in its progress, can have ocular involvement due to direct infiltration or secondary to hematological alterations typical of the disease. These findings are consistent with an involvement of the central nervous system and are thus related to the prognosis. Despite the existing systemic therapies, there needs to be more literature that shows the treatment in the ocular involvement of this disease. A case report of a child with ocular involvement due to treatment-refractory acute lymphoblastic leukemia, successfully managed with intravitreal methotrexate, is presented.

## Introduction

Leukemias are well known to be a hematopoietic stem cell neoplasm that results from abnormal proliferation and accumulation of immature lymphoid cells in the bone marrow. It is the most common childhood cancer, representing one-third of pediatric cancers. Depending on their time of presentation, they are classified as acute or chronic, and according to the predominant proliferating cell type, they can be lymphoblastic or lymphocytic.

Ocular involvement may occur due to direct infiltration or secondary to hematological alterations typical of the disease. Anterior chamber reaction increased intraocular pressure, and pseudohypopyon is an important sign of anterior chamber involvement. These findings are consistent with an involvement of the central nervous system and are thus related to the prognosis. Despite the existing systemic therapies, such as chemotherapy and radiotherapy, there needs to be more literature that shows the treatment in the ocular involvement of this disease. A case report of a child with ocular involvement due to treatment-refractory acute lymphoblastic leukemia, successfully managed with intravitreal methotrexate, is presented.

## Case report

A 4-year-old boy was diagnosed with B-cell acute lymphoblastic leukemia in January 2019 with intramedullary and central nervous system involvement, and received systemic and intrathecal chemotherapy. Subsequently, in August 2020, he suffered his first relapse and was taken again into systemic and intrathecal chemotherapy. Then, he underwent a bone marrow transplant in January 2021. Four months later, he had his second relapse with medullar, testicular, and central nervous system compromise. Due to the poor response to the treatment, a multidisciplinary staff was formed, and they decided to give palliative oral therapy.

Later on, the patient presented a marked clinical improvement despite poor treatment adherence, which was explained by a graft-versus-leukemia effect. Owing to the above, locoregional testicular and central nervous system radiotherapy, intrathecal chemotherapy, and an orchidectomy were performed. On December 2022, liver function tests were altered, so they made a liver biopsy that revealed a graft-versus-host disease. The patient was discharged with oral steroids.

**Figure 1:**
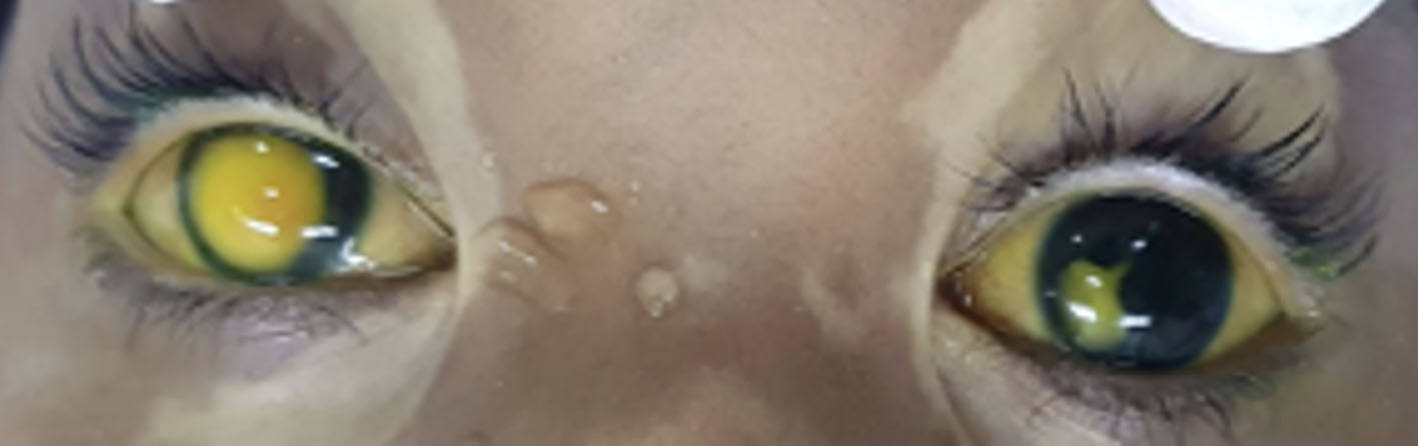
Before anterior chamber washout and intravitreal methotrexate, bilateral pseudohypopyon.

**Figure 2:**
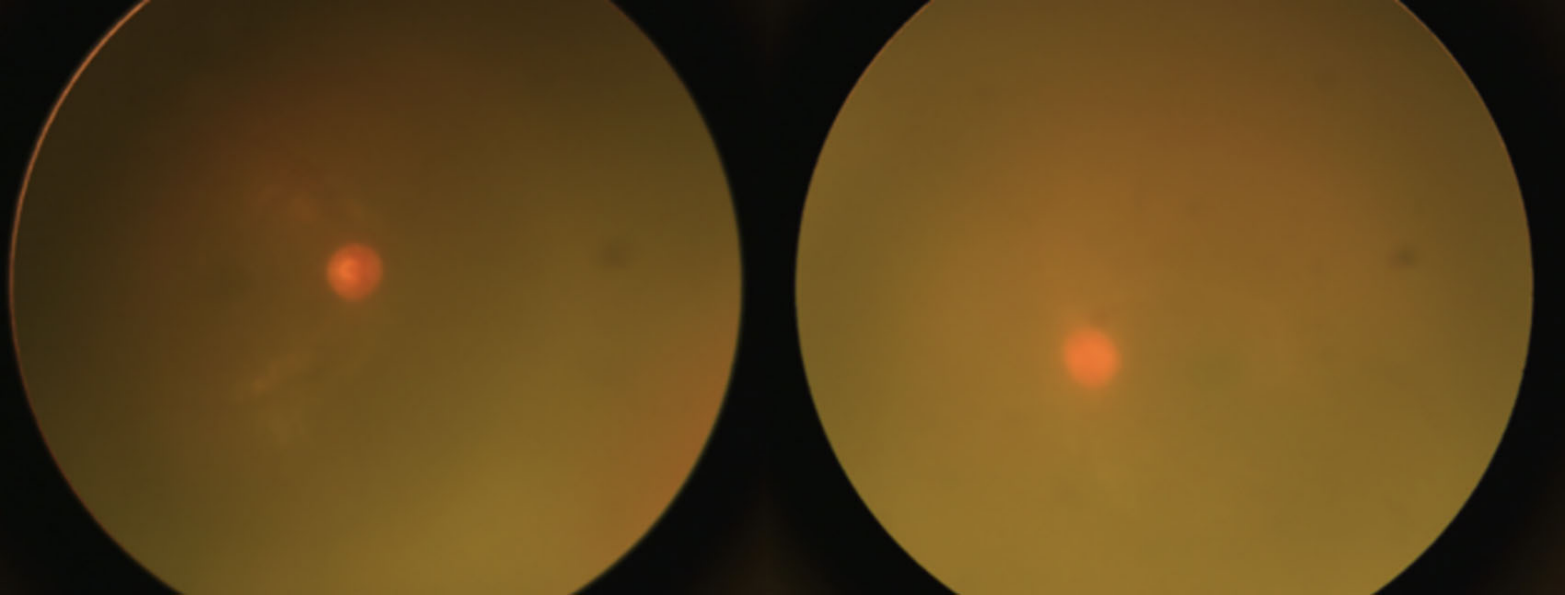
Before intravitreal methotrexate, bilateral vitreous haze.

He complained of 4 days of severe eye pain, photophobia, and a whitish lesion on the right eye in January 2022. On ocular examination, bilateral pseudohypopyon, elevated digital intraocular pressure (IOP) in the right eye, and vitreous haze on both eyes were found. Because of poor patient cooperation due to age, pain, and photophobia, it was not possible to measure the visual acuity and to find details on the slit lamp, so a physical examination under general anesthesia was performed, finding a pseudohypopyon of 4 mm and 1 mm on the right and left eyes, respectively (**Figure 1**), an IOP of 30 mmHg in the right eye and 14 mmHg in the left eye, and vitreous haze on both eyes (**Figure 2**).

Treatment was started with Dorzolamide/Timolol every 12 h, Prednisolone five times a day, and Tropicamide every 12 h, but he did not improve. Despite being a patient in palliative management, his ocular complaint was the only thing that compromised his quality of life. Therefore, the interinstitutional and multidisciplinary medical board decided to perform an anterior chamber washout and intravitreal injection of methotrexate.

**Figure 3:**
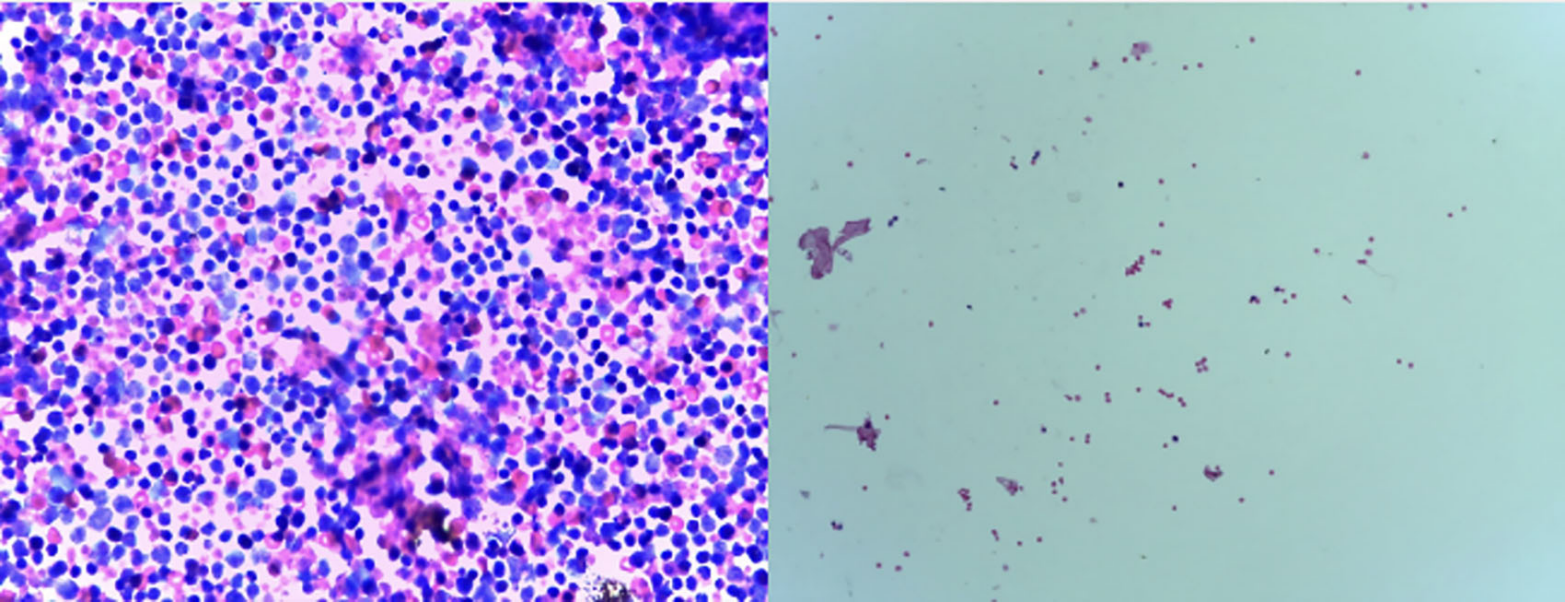
Left, aqueous humor biopsy before intravitreal methotrexate that reported atypical immature cells compatible with blasts, >500 blasts per high-power field. Right, aqueous humor biopsy after intravitreal methotrexate, five blasts per high-power field at the third week of treatment.

Anterior chamber washout was performed 9 days later to confirm the diagnosis, finding a marked growth of pseudohypopyon in both eyes. In the aqueous humor biopsy, >500 blasts were reported per high-power field (**Figure 3**). An intravitreal injection of MTX 400 μg in 0.1 mL was performed. One weekly injection for four doses was administered, and a sample of aqueous humor biopsy was taken at every opportunity under general anesthesia because of the age and the patient’s comorbidities.

**Figure 4:**
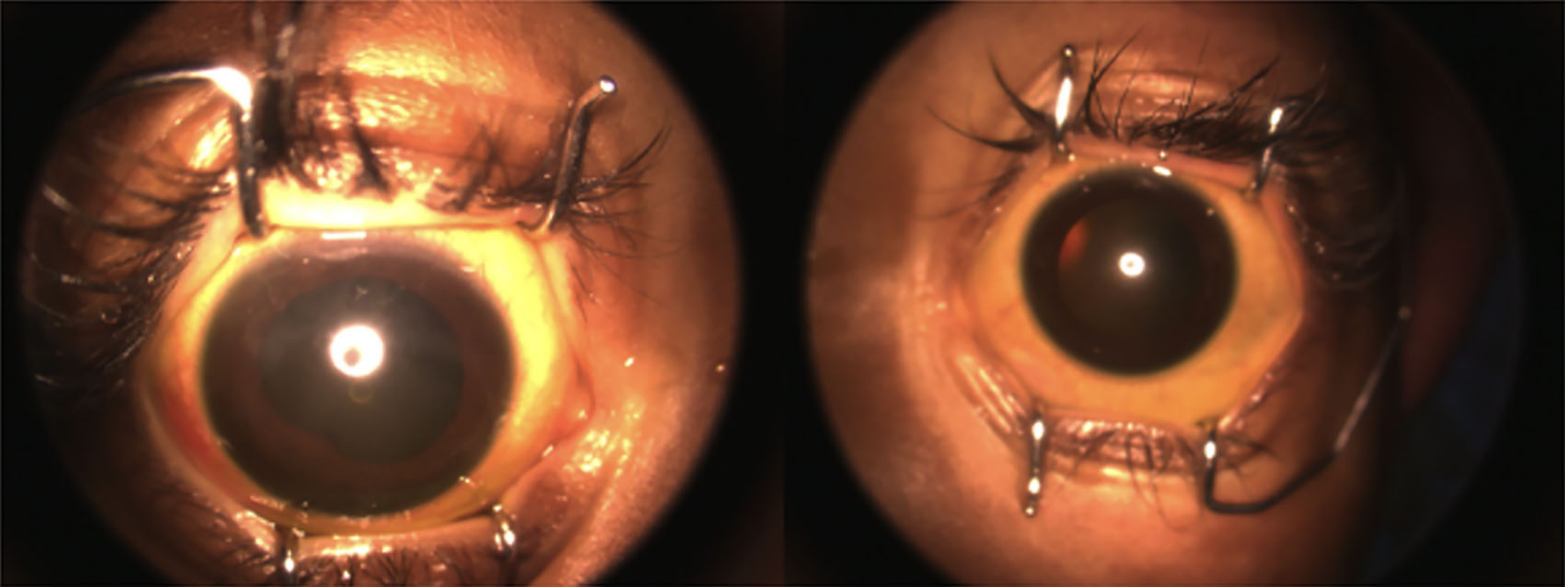
After anterior chamber washout and intravitreal methotrexate, no pseudohypopyon.

**Figure 5:**
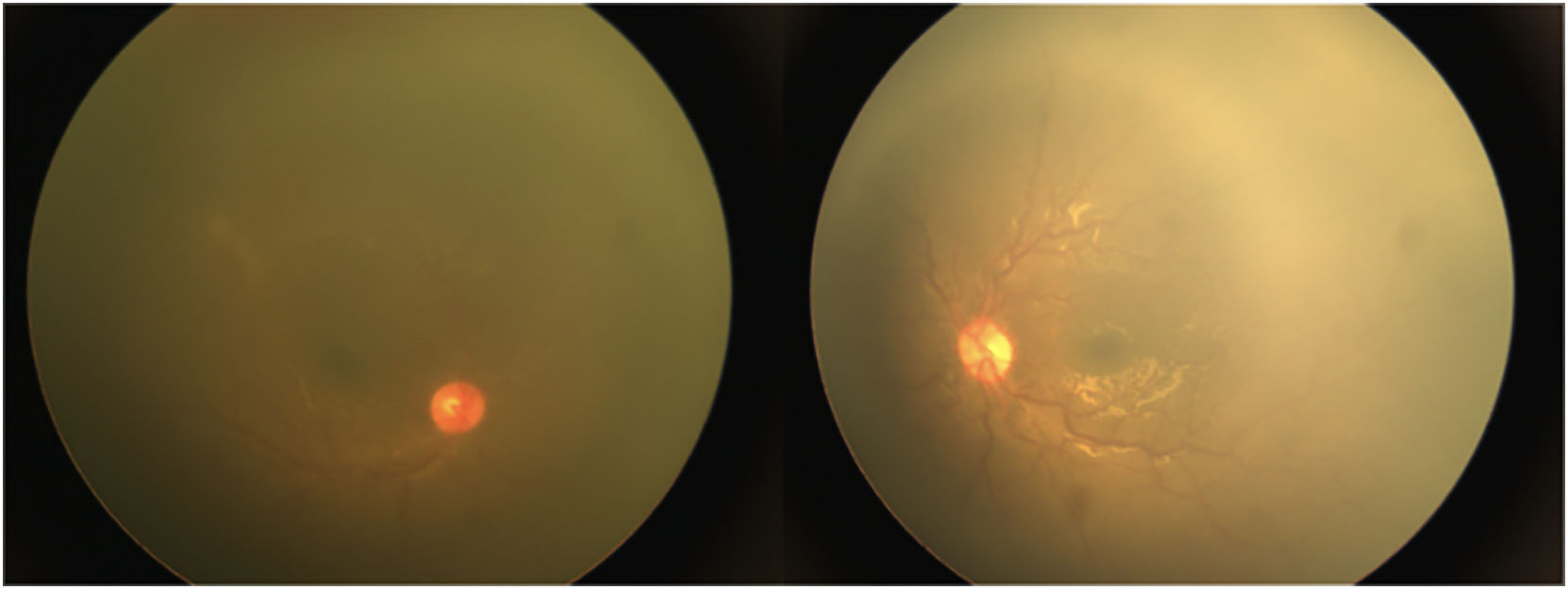
After anterior chamber washout and intravitreal methotrexate. Improvement of the bilateral vitreous haze.

In the first week after starting treatment, the patient presented a significant improvement in pain and visual symptoms, improving his interaction with the environment. On ophthalmological examination, there was no pseudohypopyon (**Figure 4**), a marked decrease in IOP, and a progressive decrease in the vitreous haze (**Figure 5**) until the fundus could be visualized without evidence of lesions. With a reduction in topical steroids and total removal of antiglaucoma and cycloplegic drugs. At the end of the treatment, an appointment was made for review. However, relapse in the CNS and peripheral blood was confirmed again with cerebrospinal fluid blasts, and the patient died 15 days after the last therapy without current clinical signs of ocular relapse.

**Figure 6:**
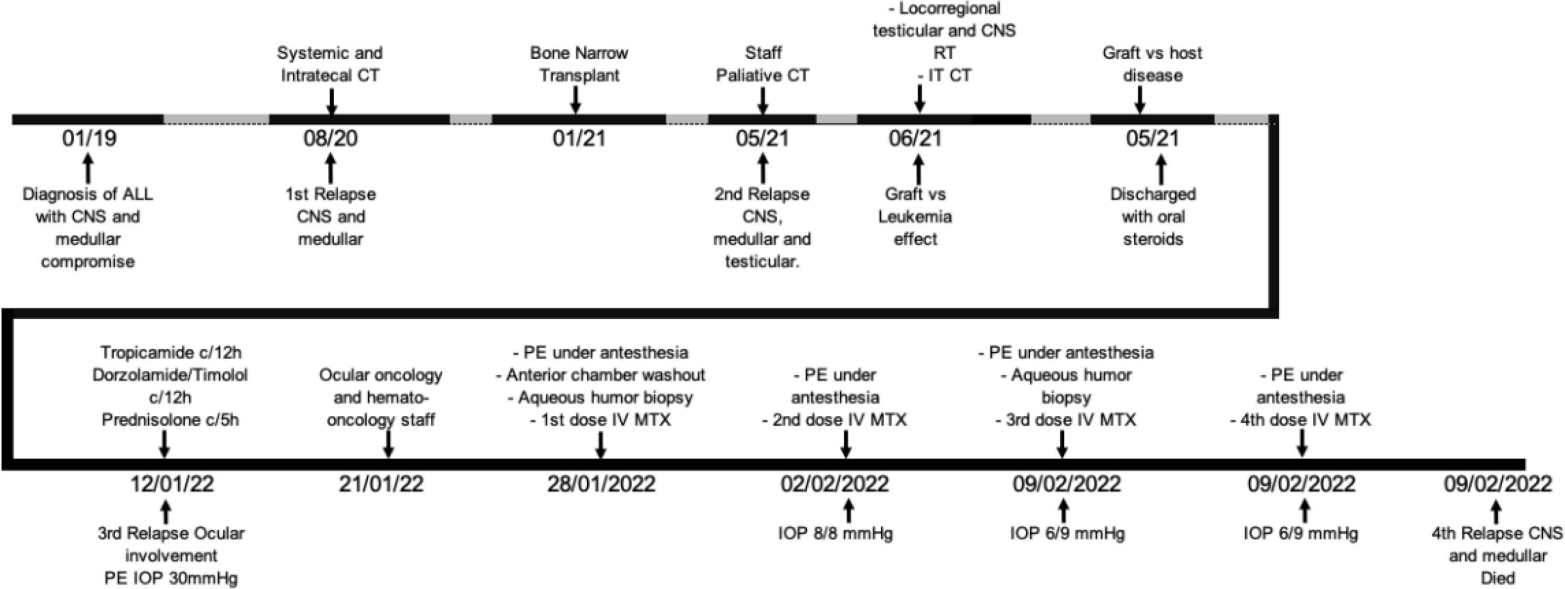
Treatment timeline.

A timeline is presented in **Figure 6**.

## Discussion

Leukemia is a group of malignant neoplasms of hematopoietic cells classified as myeloid and lymphoid or acute and chronic, depending on the cell type and its evolution. The ophthalmological manifestations of leukemia can be primary, finding leukemic infiltrates or retinal hemorrhages, or secondary with dysplastic cells or complications related to leukemia itself (1). These manifestations have been reported between 9% and 90% of patients, defining a possible invasion of the central nervous system (2). Of the patients, 2.5%–18% have compromised anterior chamber, presenting with pseudohypopyon or secondary glaucoma by deposition of lymphoblasts (3, 4). In addition, it is associated with testicular and spinal cord relapse (5).

Treatment for ocular relapse has been limited to systemic chemotherapy, biologics, and local radiotherapy. However, these strategies are insufficient or even favor the development of local ophthalmological or orbital complications. Intravitreal methotrexate has been described in the literature as a local treatment in patients with ocular involvement of acute lymphoblastic leukemia, showing an improvement in the inflammatory reaction and regression of infiltration by tumor cells even in the anterior chamber (1, 6, 7).

In addition, two cases of patients with acute lymphoblastic leukemia and vitreoretinal infiltration, which were treated with this same drug, were reported, showing a reduction in retinal infiltration and even improvement in the visual function of one of the eyes, and another patient with regression of the disease but without significant improvement in vision (8). Among the main adverse effects described in the literature are keratopathy and transient elevations in intraocular pressure.

In this case report, a child with advanced disease and palliative treatment presented a disabling ocular infiltration and severe compromise of his quality of life. After lavage of the anterior chamber and application of four doses (1 weekly) of intravitreal methotrexate, complete resolution of symptoms, decreased intraocular pressure, and regression of inflammation and vitreous compromise were achieved. Two weeks after the last dose, the patient died due to severe systemic compromise.

In conclusion, the use of intravitreal methotrexate could be considered in patients with ocular compromise secondary to acute lymphoblastic leukemia, achieving regression of infiltrates, improvement of inflammation, and symptomatic control. Additional evidence is required to safely assess this drug’s effectiveness and safety in leukemia patients.

## Data availability statement

The original contributions presented in the study are included in the article/supplementary material. Further inquiries can be directed to the corresponding author.

## Ethics statement

Written informed consent was obtained from the individual(s), and minor(s)’ legal guardian/next of kin, for the publication of any potentially identifiable images or data included in this article.

## Author contributions

SL, SM and MN have contributed in the conception, the acquisition and analysis of the work. MOP, MA, MLP and MB have contributed providing important intellectual content and made approval for publication of the content.
